# Regulatory Role of Immune Cell-Derived Extracellular Vesicles in Cancer: The Message Is in the Envelope

**DOI:** 10.3389/fimmu.2020.01525

**Published:** 2020-07-16

**Authors:** Chi Li, Howard Donninger, John Eaton, Kavitha Yaddanapudi

**Affiliations:** ^1^Experimental Therapeutics Group, James Graham Brown Cancer Center, University of Louisville, Louisville, KY, United States; ^2^Department of Medicine, University of Louisville, Louisville, KY, United States; ^3^Immuno-Oncology Group, James Graham Brown Cancer Center, University of Louisville, Louisville, KY, United States; ^4^Division of Immunotherapy, Department of Surgery, University of Louisville, Louisville, KY, United States; ^5^Department of Microbiology and Immunology, University of Louisville, Louisville, KY, United States

**Keywords:** extracellular vesicles, exosomes, nanovesicles, immune cells, cancer, immunotherapy, immune suppression

## Abstract

Extracellular vesicles (EVs) are a heterogenous group of membrane-surrounded structures. Besides serving as a harbor for the unwanted material exocytosed by cells, EVs play a critical role in conveying intact protein, genetic, and lipid contents that are important for intercellular communication. EVs, broadly comprised of microvesicles and exosomes, are released to the extracellular environment from nearly all cells either via shedding from the plasma membrane or by originating from the endosomal system. Exosomes are 40–150 nm, endosome-derived small EVs (sEVs) that are released by cells into the extracellular environment. This review focuses on the biological properties of immune cell-derived sEVs, including composition and cellular targeting and mechanisms by which these immune cell-derived sEVs influence tumor immunity either by suppressing or promoting tumor growth, are discussed. The final section of this review discusses how the biological properties of immune cell-derived sEVs can be manipulated to improve their immunogenicity.

## Introduction

In the first marathon, run solo by Philippides, both the messenger and the message were targeted (although sadly the messenger died having delivered the message). Such precision targeting would be a desirable feature for successful cancer immunotherapy and here we argue that small vesicles spontaneously released by cells might be just the ticket.

Extracellular vesicles (EVs) are cell-derived nanovesicles that exhibit immunomodulatory properties and show promise as potential therapeutic agents (1, 2). *In vitro* and *in vivo* studies suggest that cell to cell communication is facilitated via an acellular EV-mediated process, leading to intercellular transfer of molecules (3). Importantly, EVs can transfer proteins, mRNA, and microRNA, thus, facilitating the genetic exchange between cells (4). Despite significant strides made in delineating biogenesis (5) and protein/lipid composition (6), the *in vivo* biological relevance of EVs in cancer-bearing hosts remains largely unclear. Early pre-clinical studies provide evidence that EVs can operate as therapeutic agents. EVs derived from antigen presenting cells (APCs) that are loaded with either peptide or whole protein antigens are reported to induce anti-tumor immunity in animal models but show only modest improvements in cancer patients (2, 7–9). These observations support the proposal that nano-sized EVs can be used as carriers to deliver soluble antigens in tumor models (10). The currently expanding knowledge about the biological effects of EVs provides clues about the pros and cons of using EVs in cancer therapy. The initial part of this review focuses on the nomenclature and biogenesis of EVs. The initial part of this review describes the composition and mechanisms by which immune cell-derived EVs interact with and influence host cells. The final part of this review describes how the biological properties of these immune cell-derived EVs can be engineered to amplify their immunogenicity as novel anti-cancer immunotherapeutic agents.

## Nomenclature of Extracellular Vesicles (EVs)

EVs is an umbrella term that encompasses different types of vesicles including microparticles and exosomes released from eukaryotic cells. Accumulating evidence suggests that cells release EVs of different sizes and subcellular origin. The heterogeneity of EVs and the existence of non-vesicular extracellular nanoparticles creates confusion with respect to nomenclature. This also increases the complexity of defining the composition and functional properties of these very diverse secreted components. Until recently, parameters such as size, presence of unique proteins, subcellular origin, and isolation techniques that have been used to characterize the different vesicles have led to confusion rather than clarity in the field. One such example is the finding that EVs originating from late endosomes (exosomes) and vesicles originating from the plasma membrane (ectosomes/microparticles) (11, 12) share common molecular signatures and markers [e.g., TSG101and Alix (1, 13)]. In 2018, the *International Society on Extracellular Vesicles (ISEV)* endorsed EV as the generic term to be used for particles of any cellular origin that lack a nucleus and are delimited by a lipid bilayer (14). Additionally, the ISEV documented the “Minimal Information for Studies of Extracellular Vesicles (MISEV) guidelines” (15); additional findings have led to more recent updates to these guidelines (14). To counter the existing contradictions in the field of EVs, these guidelines recommend critical experimentation and reporting requirements pertaining to EV isolation, composition, characterization, and functional studies. One such class of characterization parameters include: (1) Size of EVs—small EVs (100–200 nm), large EVs (200–1,000 nm); (2) Sedimentation or density of EVs—low, middle, or high; (3) Marker expression—e.g., CD63, CD81, or Annexin A1-expressing EVs; (4) Types of cells—e.g., EVs-derived from heat-stressed cells, immune cells, apoptotic cells or hypoxic tumor cells; and (5) Biogenesis—e.g., plasma membrane or endosome.

Exosomes are 40–150 nm, endosome-derived small EVs that are released by cells into the extracellular environment. This process involves the fusion of endosomes with the plasma membrane (1). In contrast to exosomes (small EVs), microvesicles are large EVs (lEVs) and are generated via a process of shedding from the plasma membrane (16, 17).

## Biogenesis of Exosomes

Exosomes are small EVs (sEVs). sEVs are formed intracellularly by inward budding of the endosomal membrane resulting in sequestration of RNA, DNA, proteins, and lipids into intraluminal vesicles (ILVs) within the lumen of multivesicular bodies (MVBs) (17). Fusion of MVBs with the plasma membrane leads to release of ILVs which are then termed sEVs; this budding event during sEV formation occurs in a reverse membrane orientation (17). Little is known about the molecules and the cytosolic machinery involved in the modulation of the sEV secretion.

The release of sEVs into the extracellular milieu involves fusion of the MVB with the plasma membrane. Several proteins loaded into sEVs originate from the MVB membrane. Some of these proteins include the major histocompatibility complex (MHC) and costimulatory molecules that ultimately participate in sEV-mediated regulation of immune responses. The cargoes of sEVs originate from the golgi apparatus or from the plasma membrane and are sorted into MVBs before being released as ILVs. This implies that cargoes that recycle back to the plasma membrane are typically not enriched in vesicles, however, a disruption to this recycling process can occur as seen in the case of transferrin receptor in reticulocytes (18). Also, the transport of cargo from golgi to endosomes implies that any disruption to the endosomal recycling or conditions that allow “retrograde transport” from endosomes to golgi will adversely affect the sorting of cargoes into sEVs (17).

Several sorting machineries participate in successful generation of sEVs (17). In an initial step for sEV generation, membrane-associated proteins and lipids are clustered in distinct microdomains of the limiting membrane of the MVB. The process of sorting proteins into MVBs is facilitated by a set of proteins called “endosomal sorting complex required for transport (ESCRT).” The process of binding of the cargo to the endosomes is initiated by ESCRT-0. Tamai et al. demonstrated convincingly that *hepatocyte growth factor-regulated tyrosine kinase substrate* (HRS; an ESCRT-0 protein) is required for sEV generation and release (19). Following this initiation by ESCRT-0, ESCRTs-I, -II, and -III complexes act in tandem to help the cargo accumulate on the endosomal membrane (19). VPS4, an AAA-type ATPase, disrupts the ESCRT complexes and the membrane with its cargo then gets embedded into the endosome to produce an MVB (19). MVBs utilize the intercellular membrane traffic system that aids in the release of ILVs as sEVs by direct fusion with the plasma membrane (5). Members of the Rab GTPase family (Rab5, Rab11, Rab27a, and Rab27b) (20, 21) have been shown to be involved in the secretion of sEVs (22, 23). Recent research suggests that the different cellular pathways involved in sEV biogenesis depend on the properties of the producing cell. For example, sEV biogenesis in T cells occurs at the plasma membrane and exploits a unique molecular machinery that is typically associated with endosomal biogenesis of ILVs (13, 20). Additionally, sEV generation and cargo composition can be altered by interfering with the components of the ESCRT pathway. One such approach is to target ESCRT accessory protein ALG-2 interacting protein X (ALIX) (21) to disrupt the release of ILVs. This is based on the fact that ALIX protein is involved in bridging protein cargoes with other subunits of the ESCRT protein sorting machinery (21). Such methods can be utilized to engineer cargoes to be secreted as sEVs.

sEVs can also be generated and secreted in an ESCRT-independent manner (24). Recent studies have revealed that ILVs loaded with CD63 can be formed even when the functional components of the four ESCRT complexes are depleted (24). One such ESCRT-independent mechanism for generation of sEVs is dependent on ceramide (23). Ceramide, which is generated by a type II sphingomyelinase, induces the formation of membrane microdomains. Ceramide has been shown to be converted to sphingosine 1-phosphate, which then activates Gi-protein-coupled sphingosine 1-phosphate receptor, a key player that sorts cargoes into exosomal ILVs (25). One other ESCRT-independent mechanism involves proteins of the tetraspanin family (CD81, CD82, CD9, and CD63) (5, 26, 27). These proteins route various sEV cargoes by forming clusters with each other as well as with other proteins (both transmembrane and cytosolic). They also facilitate the formation of microdomains that eventually bud into cargo carrying sEVs (5, 26, 27). For example, cone-shaped clusters of tetraspanin CD81 induce inward budding of microdomains enriched with CD81 protein (28). Based on these studies, it is apparent that the transmembrane cargoes can be sorted into the sEVs via both ESCRT-dependent and -independent mechanisms. For example, sorting of MHC Class II molecules into immune cell-derived sEVs relies on both ESCRT-dependent and ESCRT-independent mechanisms. Several different cytosolic proteins become part of the cargo protein repertoire. Chaperones such as heat shock proteins and heat shock cognate proteins (HSP70 and HSC70) play an important role in co-sorting these cytosolic proteins into ILVs in cells, including immune cells (29, 30).

Additional details and other characteristics of sEVs (exosomes) have been reviewed elsewhere (16, 17, 31, 32). The following sections are focused on composition and functional properties of sEVs derived from various immune cell types that exhibit either immune stimulatory or immune suppressive functionality in cancer.

## Composition of Immune Cell-derived sEVs

Immune cell-derived sEVs contain ubiquitous and cell type-specific proteins that are involved in both immune stimulatory and suppressive functions (3, 33). Various cargoes including lipids and nucleic acids are selectively incorporated into sEVs (17). Moreover, several proteins present in the sEVs are conserved in the different parent cell types (3). A recent study by Jeppesen et al. shows that sEVs are devoid of any cytosolic glycolytic enzymes, cytoskeletal proteins, or double-stranded DNA (34). This study also identifies Annexin A1 as a specific marker of microvesicles (34).

With regards to composition and function, sEVs from antigen-presenting cells (APCs), which include Dendritic cells (DCs) and B cells, have been well-characterized. These APC-derived sEVs participate in antigen presentation via MHC Class I and MHC Class II molecules that are loaded with antigenic peptides. Antigens involved in this process are typically tubulin, actin, signal transduction protein kinases, metabolic enzymes, and heat shock proteins (3, 35, 36). Immune cell-derived sEVs are enriched in tetraspanins, a family of ubiquitous proteins, which include CD9, CD63, CD81, and CD82. These tetraspanins on sEVs have been shown to interact with other proteins such as MHC molecules and integrins expressed on the target cells, consequently leading to organization of membrane subdomains (37, 38).

Besides proteins, sEVs released from immune cells also exhibit unique lipid composition profiles. Sphingomyelin, phosphatidylcholine, and phosphatidylethanolamine are some of the lipids that are present in significant quantities in these sEVs (6, 39, 40). Additionally, sphingomyelin, cholesterol, and other lipids present in immune cell-derived sEVs are critical for the maintenance of rafts (41). sEVs mediate exchange of lipids between different cells. In contrast to lipoproteins that contain a phospholipid monolayer, sEVs have a bilayer membrane surrounding cytosolic material and therefore transport lipids using a distinct mechanism. These sEVs display increased “trans-bilayer flip-flop movements of phospholipids.” These flip-flop movements facilitate the fusion of sEVs to target cell membrane (6). Immune cell-derived sEVs also harbor a unique set of enzymes that participate in lipid metabolism (phospholipase A2, C, and D) (39, 42). Intriguingly, immune cell-derived sEVs possess a phospholipid composition that is different from that found in the parent cells. DC-derived sEVs (dexosomes) have higher sphingomyelin and lower phosphatidylcholine levels when compared to that observed in the parent cells. Phospholipase D2 is highly expressed in sEVs and this enrichment allows these vesicles to interact with target cells. This process is dependent on the fusogenic properties of its signaling messenger—phosphatidic acid (PA) (43). PA can trigger membrane fusion in the presence of calcium (44) that subsequently facilitates interdigitation of lipid molecules between different membranes (41).

Recent technological advances such as next generation sequencing have paved the way for identification of the nucleic acid cargo (DNA and RNA) harbored in sEVs (4, 45). Immune cell-derived sEVs have been shown to harbor miRNAs that influence both innate (monocytes) and adaptive immunity (T and B cells) in cancer. sEVs also harbor long non-coding (lnc) RNAs that are then transferred into target cells (46). Several different lncRNAs (MALAT-1, linc-POU3F3, ZFAS1, and GAS5) have been identified (47–49). sEVs harboring GAS5 are also known to regulate innate and adaptive immune responses (50–52). A better understanding of the molecular events within the parent cell that are involved in the sorting and processing of miRNA and lncRNA into sEVs is key toward developing novel biomarkers and sEV-based therapeutics.

Additional details of the cargo that participate in the immune stimulatory or suppressive functions of the immune cell-derived sEVs are discussed below.

## Isolation of sEVs and Their Interaction With Target Cells

Differential ultracentrifugation is a commonly employed procedure used to isolate sEVs released into the extracellular milieu. One limitation of this widely used procedure is that it does not allow discriminating between sEVs and other vesicle-like structures. This limitation can be overcome by using sucrose gradient floatation (53). Since sEVs float on sucrose gradients, sEVs can be separated from other contaminants. However, repeated ultracentrifugation can induce irreversible damage to the vesicles and reduce yields (54). An alternative to ultracentrifugation is concentration of sEV-containing solution using ultrafiltration devices or size-exclusion chromatography (53). Particle yield and purity depend on the type of isolation method used and the source of the sEV-containing sample (e.g., sEV isolation from cell-culture supernatant vs. sEVs from human plasma). Robust and high-throughput methods that maximize isolation of homogeneous sEVs are critical to establish biological applications involving sEVs. In a recent study, Robert Coffey's group has employed a two-step method to isolate pure sEVs: step (1) high-resolution iodixanol density gradient fractionation to separate sEVs from other contaminating vesicles; followed by step (2) direct immunoaffinity capture (DIC) with capture beads targeting sEV tetraspanins (34). This two-step method allows for the separation of a low-density fraction (cup-shaped vesicles with morphology and size consistent with sEVs) and a high-density fraction (pool of non-vesicular components) (34).

sEVs in the extracellular milieu can transfer their contents to a target cell and influence its function and phenotype. This transfer process involves a sequence of events that include docking of sEVs at the plasma membrane, surface receptor activation/signaling by the target cell, and endocytosis of the vesicle or its fusion with target cells. Details of the molecular events that are part of this delivery process, including uptake and intercellular trafficking, are yet to be unraveled. The events are likely to rely on multiple variables such as the origin of sEVs as well as identity of recipient cells.

It is very likely that the recognition between sEVs and the target cell involve proteins present at the cell surface of sEVs (55, 56). Immune cell-derived sEVs have been shown to communicate with target cells via expression of a series of cell-specific transmembrane proteins (e.g., DC-specific α and β chains of integrins, B cell-specific ICAM-1 and CD54 proteins), cells surface peptidases [e.g., aminopeptidase N (CD13) expressed on mast cells], and the integrin-interacting protein lactadherin (3, 35, 57, 58). Tetraspanins expressed on sEVs have also been reported to interact with integrins and facilitate sEV uptake by target cells (59–62). CD44 is a cell surface glycoprotein expressed on immune cells and has been reported to be involved in docking and uptake of sEVs (63). Cargoes delivered by sEVs can elicit signaling events in the target cells. These events have been well-documented for sEVs derived from B cells and dexosomes which when internalized are able to present antigens to T cells and activate antigen-specific T cell-mediated immune responses (8, 64). Cargoes released from sEVs into target immune cells (including APCs) are processed in the endocytic compartment in a manner similar to that used for processing other internalized antigens. Thus, cargoes delivered by sEVs can directly influence immune modulatory functions of the target cells (55) (discussed further in the sections below).

## Immunostimulatory Effects of Immune Cell-derived sEVs in Cancer

**Figure 1:**
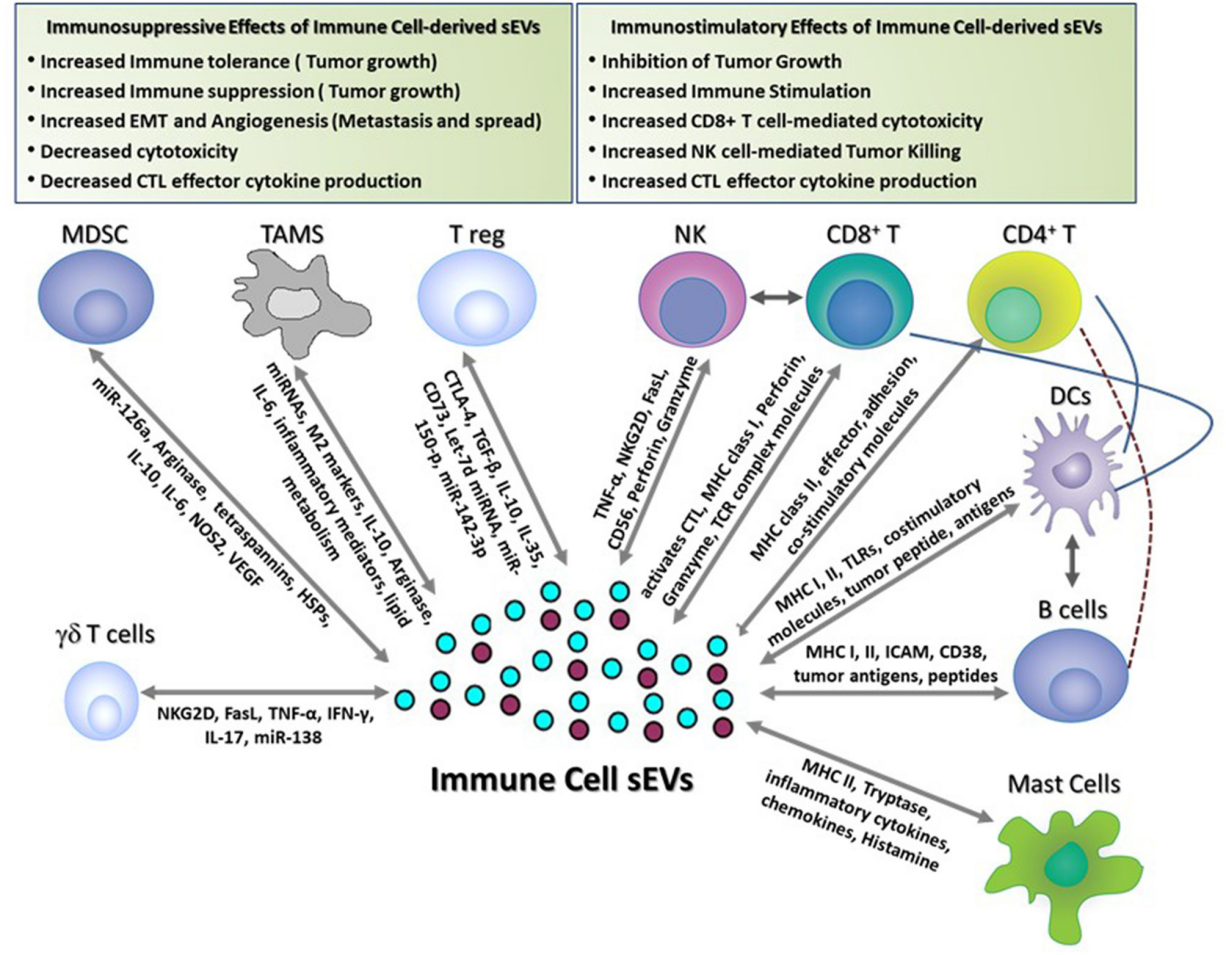
Immune-stimulatory and suppressive effects of immune cell-derived sEVs in cancer. This figure shows the different functions of immune cell-derived sEVs that have been reported to play a role in modulating immune responses in tumor-bearing hosts. These sEVs activate anti-tumor immune responses and inhibit tumor growth by (1) inducing Dendritic cell- and B cell-mediated antigen presentation, (2) promoting CD4^+^ T cell helper responses, (3) activating CD8^+^ T cell- and NK cell-mediated effector responses and cytotoxicity, and (4) inducing mast cell-mediated secretion of immune regulatory cytokines and chemokines. Conversely, immune cell-derived sEVs suppress the immune responses and promote tumor growth, invasion, and metastasis by (1) suppressing T cell-mediated anti-tumor effector responses and cytotoxicity, (2) promoting angiogenesis and EMT, (3) inducing FasL-mediated T cell apoptosis, and (4) producing immune suppressive soluble mediators and cytokines.

Exosomal sEVs derived from DCs, B cells, T cells, NK cells, and mastocytes have been widely characterized and are known to exhibit diverse functional properties (Figure 1). In the case of sEVs derived from APCs (B cells and DCs), immune stimulation requires interaction of antigen-loaded MHC-I and MHC-II molecules expressed on sEVs with TCRs expressed on T cells. Alternatively, T cell stimulation may be via an indirect route that mainly occurs through a process of internalization of antigen-loaded exosomes by APCs that then present the processed antigen as MHC-restricted peptides to T cells. APC-derived sEVs carry pro-inflammatory cytokines and chemokines that can influence T cell migration and expansion. Apart from antigen presentation, sEVs isolated from immune cells such as NK cells express cytolytic proteins and granules that can perform effector functions and induce cytotoxic cell lysis and elimination of tumors and, in some cases, activated immune cells. Likewise, CD8^+^ CTL-derived sEVs can produce cytotoxic cytokines and can target tumor cells for cytotoxic elimination. Mastocyte-derived sEVs are known to carry effector cytokines IL-2 and IFN-γ, induce B cell and T cell proliferation, and induce DC maturity (65). Cumulative research over the last decade suggest that sEV-mediated immune stimulation is dependent on functional interactions between several different immune cell populations. For example, induction of CD8^+^ T cell-mediated anti-tumor immunity by B cell-derived sEVs is reliant on cross-talk with the T helper cell arm (CD4^+^ T cell) and innate cell arm (NK cells) of the immune system (66). Along similar lines, sEVs from DCs loaded with proteins (but not peptides) are efficient activators of the immune responses and this immune activation is dependent on help from CD4^+^ T cells and B cells (67). In this section, the immune stimulatory properties (composition of cargo, target cell recognition and cargo release) of sEVs derived from immune cell types (mentioned above) are discussed in detail (sections Dendritic Cell-Derived sEVs, B Cell-Derived sEVs, Natural Killer (NK) Cell-Derived sEVs, T Cell-Derived sEVs, and Mast Cell-Derived sEVs).

### Dendritic Cell-Derived sEVs

Dexosomes (sEVs released from Dendritic cells) have received attention as anti-cancer immunotherapeutic agents since they harbor functional immune activating cargo (68, 69). Dendritic cells (DCs) are attracted to the site of dying tumor cells and play a key role in driving tumor antigen-specific T cell-mediated anti-tumor responses. The stability, ease of storage/shipping and more efficient uptake into target cells (when compared to that of soluble molecules) make dexosome-based vaccines a viable therapeutic strategy against cancer (7, 8, 10, 70). Immunostimulatory effects of dexosomes have been tested in pre-clinical mouse and *ex vivo* human models of cancer as well as in clinical trials. Dexosomes loaded with α-fetoprotein [AFP; (71)] are effective against hepatocellular carcinoma in mice. Immunization with AFP-containing sEVs results in a Th1-mediated anti-tumor CD8^+^ T cell infiltration into the tumors that is accompanied with a significant reduction in intra-tumoral T regulatory cells (71). In another example, mice immunized with sEVs from DCs loaded with cervical cancer-associated antigenic peptide (HPV early antigen 7 peptide) were protected against cervical cancer (72). Murine dexosomes have been shown to be able to stimulate melanoma antigen-specific CD4^+^ and CD8^+^ T cells *in vitro* and generate anti-cancer immunity *in vivo* (8, 73–75). In a HLA-A2 transgenic mouse model, sEVs pulsed with tumor peptides efficiently prime MART1-specific CTLs (76). Murine dexosomes indirectly loaded with either tumor cell lysate or total tumor RNA have been tested against aggressive tumors that respond poorly to therapies involving single tumor antigens. DC-derived sEVs loaded with tumor lysate enriched with molecular chaperone family of proteins such as calreticulin and heat shock protein 70 and 90 are reported to present a superior source of tumor antigens and immune responses *in vivo* than that offered with sEVs loaded with lysates obtained from freezing/thawing of tumor cells (77). DC-derived sEVs that have been indirectly loaded with a combination of common antigen ovalbumin and immunogenic invariant NK ligand α-galactosylceramide (αGC) have been shown to impart immunostimulatory effects *in vivo* in mouse models of cancer (78).

The maturation status of the DCs as well as the nature of the maturation stimuli have been shown to dictate the immunostimulatory efficacy of the DC-derived sEVs (79). DC maturation is best achieved upon treatment with TLR3, TLR4, or TLR9 ligands [poly(I:C), LPS, and CpG-B oligonucleotide; (79)]. EVs derived from DCs exposed to hyperthermia and stress (80, 81) contain increased amounts of heat shock proteins and chemokines (CCL2, CCL5, and CCL20) and as such, promote infiltration of tumor-attacking T cells and DCs into the tumor microenvironment of mice treated with these EVs (82). In addition to inducing CD4^+^ T and CD8^+^ T cell as well as NK cell-mediated responses, DC-derived antigen-loaded sEVs can induce B cell activation and IgG secretion which play an important helper role in activation of adaptive anti-tumor T cell responses (67, 75, 83, 84). Human DC-derived sEVs have been reported to be involved in activation and proliferation of NK cells and this activation process involves proinflammatory cytokines family of ligands including TNF and IL-15Rα (85, 86).

Although, in pre-clinical mouse and *ex vivo* human models of cancer, dexosome-based therapy is efficacious in inducing sufficient anti-tumor immunity, it has had no demonstrable success in clinical trials of cancer. In clinical trials with melanoma and lung cancer patients using sEVs from autologous monocyte derived-DC cultures, only modest anti-tumor responses were observed (7, 9, 87). This failure to obtain robust anti-tumor immune responses has been attributed to diminished NK cell function (9, 87). One limitation of this immunotherapeutic strategy could be that the dexosomes lack co-stimulatory signals required to mediate activation of CD8-mediated T effector cells that are equipped to recognize and destroy tumor cells. Also, the antigenic drift and a highly immune suppressive tumor microenvironment present in the non-T cell inflamed tumors could be important contributing factors for the therapeutic failure.

### B Cell-Derived sEVs

B cells are a central component of the humoral arm of the immune system and are involved in presentation of antigens and production of immunoglobulins and pro-inflammatory soluble mediators. Receptors expressed on the B cells capture extracellular antigens and toxins which are then translocated and processed via the endosomal pathway for antigen presentation or harbored into vesicles and released into the extracellular milieu (88–90). sEVs isolated from both murine and human B cells contain MHC class II molecules and human leukocyte antigens (HLA) along with co-stimulatory and adhesion molecules that are part of the antigen presentation machinery of B cells; high levels of expression of these molecules present as cargo can induce antigen-specific immunostimulatory responses against cancer (37, 64, 91). sEVs derived from B cells that are loaded with MHC class II molecules have been shown to stimulate CD4^+^ T cells *in vitro* (64). B cell-derived sEVs also harbor functional integrins that then facilitate high-affinity interactions with other cells, including cytokine-activated fibroblasts (92).

B cell-derived sEVs, akin to other immune cell-derived sEVs, interact with other immune cells including DCs, macrophages, T cells, and influence their functionality; these associations occur in different immune and non-immune compartments that include blood, spleen, lymph nodes, and tumors (93). In this context, B cell-derived sEVs are known to migrate across tissue barriers by forging interactions with extracellular matrix proteins (ECM) for delivering the cargo to recipient effector cells which involves specific receptor-ligand interactions (92). B cell-derived sEVs have been shown to harbor functional integrins and ICAM-1 that facilitate high-affinity interactions with ECM which paves way for delivery of cargo to T cells. This cargo transfer is facilitated via the interaction of ICAM-1 with leukocyte function-associated antigen (LFA)-1 molecules expressed on T cells. In some cases, B cell-derived sEVs are known to deliver cargo to pro-inflammatory cytokine-activated fibroblasts (92).

Despite the high levels of expression of MHC class I molecules, antigen-loaded B cell-derived sEV-mediated CD8^+^ T cell immunogenicity relies on its complex interactions with other immune cells including host APCs (DCs/macrophages), CD4^+^ T cells and NK cells. Data from *in vivo* mouse studies show that the antigen presentation to CD8^+^ T cells requires MHC I on host APCs and is independent of sEV MHC I (94, 95), reiterating the importance of host immune cell interactions in B cell sEV-mediated CTL induction, a key step to enhance anti-tumor immunity. Likewise, another mechanism of induction of T cell immunity can be via DC-mediated cross-presentation of B cell-derived sEV antigens to T cells *in vivo*. Saunderson et al. showed that Langerin^+^CD8α^+^ DCs residing in the spleen take part in the cross-presentation of B cell-derived sEV antigens to CD8^+^ cytotoxic T cells following immunization into mice (66).

On their surface, B cell-derived sEVs carry CD38, an active glycoprotein enzyme, that associates with signaling complexes HSC-70, Lyn, and CD81; this is akin to the associations of CD38 with signaling complexes in the membrane rafts during TCR engagement. CD38 ligation-induced signaling cascade can potentially act as intercellular messengers of T cell activation (96). Papp et al. have reported that B cells covalently fix the complement C3 fragments on their cell membrane and these fragments are then released onto the B cell-derived sEVs. Antigen-loaded B cells release C3-carrying sEVs which then interact with G protein coupled receptors on T cells. This interaction lowers the antigenic stimulation threshold required for inducing T cell activation (97). In the tumor microenvironment as well as in draining lymph nodes, pathological activation of the complement pathway and interplay between immune cells and tumor cells can lead to release of sEVs bound with C3 fragment, ensuing immune modulation. In contrast to the primary B cell-derived sEVs, several B cell-derived lymphoblastoid cell-lines have been shown to release sEVs that harbor FasL molecules. These FasL-bound sEVs are capable of inducing apoptosis in CD4^+^ T cells and dampening the host immune responses (98).

### Natural Killer (NK) Cell-Derived sEVs

NK cells are large lymphocytes that can efficiently kill oncogenic transformed tumor cells, cells that are infected with certain viruses, and cells that are devoid of MHC class I antigen expression. This innate killing activity is not governed by any antigen specific recognition, prior activation or immunization. NK cells also take part in the immune regulation of the adaptive arm of the immune system via secretion of proinflammatory cytokines and chemokines (99, 100). Seminal research has identified several different immune check points that participate in anti-tumor effector functions of NK cells (101, 102). These findings have led to the emergence of NK cells as important targets for cancer immunotherapy. NK cell-based therapeutic efficacy can be enhanced significantly by increasing its ability to home in into the tumors. The nano-size, diffusion and retention capabilities of NK cell-derived sEVs, and the leaky vasculature of the solid tumors (103, 104), provide an environment where tumor-killing effector molecules can be delivered directly to the tumor site, and thus, overcome the homing deficiency of whole NK cell-based therapies. sEVs released from human NK cells have been shown to contain prototype NK cells markers (CD56, CD16). These sEVs can induce target cell death in the following ways: interactions of death receptor ligand FasL (on sEVs) with its receptor Fas on target cells; and releasing lytic granules perforin upon fusion with the target cells. In one of the first studies, Lugini et al. showed that sEVs purified from resting human NK cells exhibit cytotoxic activity against hematologic cell lines but do not kill solid tumor cells (105). However, sEVs isolated from activated human NK cells contain higher levels of cytotoxic effector proteins granzyme A/B and perforin, thereby mediating significant *ex vivo* cytotoxic activity against a variety of solid tumor lines including neuroblastoma (106).

In an *in vivo* study with a mouse model of melanoma, intra-tumoral injection of NK cell-derived sEVs caused inhibition of tumor growth (107). These sEVs isolated from NK-92 cells express cytolytic proteins FasL, TNF-α, and granules perforin and are able to induce cytolytic activity against melanoma cells (107). In a subsequent study, NK cell-derived sEVs were tested against glioblastoma xenograft tumors in mice. In this model, NK cell-derived sEVs administered via intravenous route are able to cross the blood-brain barrier, accumulate specifically at the tumor site, persist for several days, and suppress the growth of neuroblastoma (108). NK-cell derived sEVs also participate in maintaining immune homeostasis by acting against over-reactive immune cell expansion. In fact, circulating NK cell-derived sEVs can be isolated from healthy donor plasma. Such sEVs contain classical NK markers CD56, perforin, and activating receptor NKG2D but lack FasL expression suggesting that circulating sEVs from resting NK cells can participate in immune regulation in a paracrine fashion (109). Apart from plasma membrane fusion and receptor-ligand interactions, NK cell-derived sEVs can induce target cell cytotoxicity in an activated caspase pathway-mediated or granulysin-mediated mechanisms (110).

One question that remains to be answered is: how do NK cell-derived sEVs recognize and show specificity toward tumor cells or activated immune cells? NK cells rely on a “self-missing” mechanism to recognize and kill target cells; however, it is not clear if such a phenomenon applies to NK cell-derived sEVs. One suggested mechanism is that once the sEVs reach the tumors, the acidic microenvironment promotes the fusion of sEVs with the tumor cells or with the stomal immune cells which then results in uptake and activation of sEV-mediated biological functions.

### T Cell-Derived sEVs

Activated CD8^+^ T cells are indispensable for tumor cell destruction and elimination. T cells perform these activities by direct interaction with target cells presenting various tumor antigens along with MHC class I molecules (111). In addition to tumor cell killing, activated CD8^+^ T cells are known to execute tumor cell elimination using several different indirect methods, one of which is via sEVs. CD8^+^ T cell-derived sEVs express receptors that directly recognize target cell antigens presented in the context of MHC class I at the immunological synapse, contain cytotoxic effector cytokines (e.g., IFN-γ, TNF-α), and proteins (e.g., perforin, Granzyme B) that induce target cell lysis. sEVs carrying these molecules cumulatively participate in linking the cytotoxic T cells with the target cells (tumors cells in case of cancer) and mediate CTL-mediated destruction of the target cells (72). TCR stimulation (not mitogenic stimulation) induces release of sEVs from both *in vitro* cultured T cell clones and T cell blasts circulating in the peripheral blood of healthy volunteers (112). The cargo in these endocytic sEVs are composed of TCR complex molecules (CD3ε, ζ chain, and TCR β), CD63, CD81, adhesion molecules (CD2 and LFA-1), Src family of tyrosine kinases, c-Cbl, and chemokine receptors (112). Apart from these proteins, T cell-derived sEVs harbor heat shock proteins, enolase, integrins, and proteins of antigen processing machinery (MHC class I and β2-microglobulin).

In an *in vitro* study, the authors show that sEVs from CD3^+^ T cells activated with TCR stimulation in the presence of IL-2 stimulate autologous proliferation and cytokine and chemokine production in resting T cells (113). Seo et al. recently reported that sEVs from activated T cells, when injected into the tumor microenvironment in mice, are engulfed by mesenchymal stromal cells. This activity leads to depletion of the stromal cells that include PDGFRα^+^ CD140a^+^ mesenchymal stem cells and α-SMA^+^ cancer-associated fibroblasts that ultimately results in reduction of tumor growth, invasion, and metastasis (114). CD63-expressing sEVs from T cells are reported to polarize toward the immunological synapse formed during cognate APC-T cell interactions. This interaction facilitates the unidirectional transfer of miRNA from T cell-EVs to APCs such as B cells. Such miRNAs (e.g., miR-335) play a functional role in regulating gene expression and activation in B cells (115). The sumoylated heterogeneous nuclear ribonucleoprotein A2B1 (hnRNPA2B1) binds specifically to miRNAs and is reported to control the loading of miRNAs into vesicles (116). sEVs released from activated T cells are known to carry cargo that interact with endothelial cells and play an active role in promoting angiogenesis. sEVs from activated T cells carrying miR-142-3p upon interaction with endothelial cells induce endothelial permeability. CD47-expressing T cell-derived sEVs are reported to induce VEGF-dependent angiogenic activity by promoting endothelial cell expansion and tube formation (117).

Much like CD8^+^ T cells, CD4^+^ T helper cells activated by TCR stimulation in the presence of co-stimulatory signals release plasma membrane sEVs into immune synapses and exhibit immune regulatory properties (112, 118). van der Vlist et al. used a flow cytometry-based method to analyze sEVs. They find that a heterogenous pool of sEVs (based on density fractionation) are released from activated CD4^+^ T cells. This heterogeneity relies on the strengths of TCR activation and costimulatory signals (119). In fact, resting CD4^+^ T cells preferentially uptake sEVs released from circulating IL-2 activated CD4^+^ T cells. This process can induce proliferation and expansion in these resting cells. Such an autologous cellular tropism of circulating CD4^+^ T cell-derived sEVs can have important implications in disease conditions such as cancer and HIV (120). Along similar lines, mitogen-stimulated CD4^+^ T cells take up OVA-MHC Class I^+^ DC-derived sEVs and express acquired molecules MHC class I and OVA. These modified T cells can perform the role of APCs and stimulate antigen-specific CD4^+^ T-dependent CD8^+^ T cell proliferation, expansion and anti-tumor effector responses *in vivo* (73, 121). In a reciprocal interaction, unilateral transfer of mitochondrial DNA from T cell-derived sEVs to DCs primes activation of the cGAS/STING/DNA-sensing immune signaling pathway and expression of IRF3-responsive interferon genes that protects the DCs from infections and other insults (122).

A recent report shows that sEVs derived from follicular T helper cells (fTh) participate in modulating B cell function and differentiation (123). Given that fTh play a vital role in the differentiation of B cells into plasma cells, fTh-derived vesicles can perform similar functions in influencing B cell differentiation and affinity maturation in cancer.

### Mast Cell-Derived sEVs

Mast cells are innate immune cells located in the mucous membrane and connective tissue. These are the major effectors involved in allergy responses. Activated mucosal and connective tissue mast cells play an important role in expulsion of parasites by releasing biologically active granules (histamine), proinflammatory lipid mediators, and Th2-type cytokines (IL-4, IL-10) (124). Mast cells express Toll like receptors (TLR) and display immune regulatory activity by producing cytokines and chemokines (IL-5, IL-6, IL-10, IL-13, and TNF-α) that influence differentiation and biological functions of adaptive immune cells (DCs, T, and B cells) (125–127). Important stimuli for activation of mast cells are evoked by synergistic allergen-mediated engagement of FcϵRI (receptors for the Ig Fc portion) and TLR signaling. This leads to activation of transcription factors that are involved in secretion of soluble mediators, cytokines, and chemokines (128). During inflammation and other pathological disease conditions, mast cells accumulate at various primary and secondary immune organ interfaces (skin, lung, gut, tonsils, and lymph nodes) and orchestrate immune responses (129, 130). It appears that mast cells induce both pro- and anti-tumorigenic responses and these functions are dictated by type and stage of cancer. Mast cells accumulate in the blood as well as in the tumor microenvironment and support tumor progression in certain cancer types. In other settings, they regulate DC and T cell functions and contribute to anti-tumor immune responses (131).

Mast cell-derived sEVs are reported to carry 200–400 different proteins, many of which are yet to be identified. Few identified proteins include Plasminogen Activator Inhibitor Type I, proinflammatory cytokines, secretory granules, α and γ subunits of FcεRI, mast cell-related G protein-coupled receptor family member X2 (MRGX2), tryptase, and MHC class II molecules which are found exclusively on the sEV membrane (132, 133). Initial research suggests that sEVs in mast cells are contained within either endosomal (type I) or secretory lysosomal compartments (type II). sEVs from type I compartment colocalize with mannose-6-phosphate receptors and lamp I/II. sEVs from type II colocalize with serotonin. sEVs within these two compartments carry different protein/mRNA cargo and thus, exhibit differential immune regulation properties (65). Later studies confirmed that, activated mast cells release sEVs through two secretory routes: constitutive secretion [e.g., mast cells pre-treated with IL-4; (65)] and exocytosis [mast cells activated via FcεRI crosslinking; (134, 135)]. Carroll-Portillo et al. (133), using immunoprecipitation and electron microscopy studies, showed that mast cell-derived sEVs carry intact FcεRI. Furthermore, “right-side out” orientation of these vesicles exposes the FcεRI–IgE complexes on their cell surface. This facilitates continued recycling of cross-linked antigens and amplification of immune response. In this context, uptake of IgE and antigen loaded mast cell-derived sEVs by APCs (B cells and DCs) can result in efficient presentation of antigenic peptides to T cells. A novel functional role of mast cell-derived sEVs is their ability to package mRNA and microRNA into their lumen and transfer these shuttle RNA (esRNA) to recipient cells that engulf these vesicles (136, 137). This can then result in initiation of protein translation in recipient cells, which in essence facilitates the intercellular communication between mast cells and other immune or tumor cells (136). Mast cell-derived sEVs were reported to carry endocytosed exogenous antigens that associate with chaperone proteins (heat shock proteins 60 and 70) and elicit adjuvant-like immunity. Also, sEVs from mast cells are capable of inducing *in vivo* IgG1- and IgG2a-mediated antibody responses. Upon exposure to mast cell-derived sEVs (but not B cell- or macrophage-derived sEVs), immature DCs upregulate maturation markers (MHC class II, CD40, CD80, CD86) and produce IL-12. These activated DCs can cross-present antigens from mast cell-derived sEVs to T cells suggesting that mast cells sEVs can induce functional activation of DCs (65). Given the plasticity of DCs, it appears that the interactions between mast cell-derived sEVs and DCs are likely to vary depending on the tissue type, e.g., skin vs. tumor microenvironment.

In addition to immune-stimulatory functionality, mast cell-derived sEVs can also promote angiogenesis and immune tolerance. Mast cell activation can lead to release of sEVs loaded with tryptase, which can induce the proliferation and migration of endothelial cells and promote angiogenesis (138). Also, sEVs released from mast cells that are pre-exposed to oxidative stress carry mRNA that are capable of inducing oxidative stress tolerance in target cells; this functional property differs from that present in sEVs obtained from non-stressed parental cells (136). In a recent study, Liang et al. used bioinformatic tools to compare and contrast the gene expression profile of mast cells and mast cell-derived sEVs. This analyses reveal that several genes are differentially expressed in mast cell-derived sEVs (139).

## Immunosuppressive Effects of Immune Cell-derived sEVs in Cancer

The tumor microenvironment is comprised not only of malignant cells, but also non-malignant cells such as immune cells, fibroblasts, and vascular and lymphatic cells. The dynamic interplay between the tumor cells and these stromal cells plays a central role in establishing an immunosuppressive microenvironment (140). While many of the immunosuppressive immune cells recruited to the tumor microenvironment mediate their suppressive function through secretion of soluble mediators, there is now emerging evidence that immune cell-derived sEVs also contribute to their immunosuppressive activity (Figure 1). Virtually every immune cell releases sEVs with cargo that represents the molecular expression of the parental cell. Secretion of immunosuppressive sEVs greatly amplifies a given cell's influence in the TME by allowing it to impact a number of target cells. Identification of the specific immunosuppressive cargo and mechanisms mediated by immune cell-derived sEVs can lead to new therapeutic targets and strategies which may improve the efficacy of anti-cancer therapy.

### sEVs-Derived From MDSCs

Myeloid-derived suppressor cells (MDSCs) are immature myeloid cells that suppress T cells and NK cells activity and dampen the tumor-killing capabilities of these cells (141). MDSCs accumulate in late-stage cancer patients and contribute significantly to immunotherapeutic resistance in cancer (141). MDSCs also contribute to immune suppression in other disease settings such as chronic infections (142). Recently, one of the areas of research in the immuno-oncology field has been focused on pharmacological inhibition of MDSC activity and combining these drugs with other known immunotherapeutic agents to improve therapeutic responses in cancer patients. A recent report suggested that common exosomal proteins (e.g., annexins, tetraspanins, cytoskeletal proteins, heat shock proteins) as well as several unique proteins are present in both MDSCs and MDSC-derived sEVs (143, 144). These sEV-derived immune suppressive proteins participate in immune regulation in cancer-bearing hosts (143, 144). MDSC-derived sEVs are also implicated in therapeutic resistance in cancer-bearing hosts (145–147). Chemotherapy induces MDSC numbers in tumor-bearing hosts that results in attenuation of the anti-cancer efficacy of the chemotherapy (148–150). Notably, in tumor-bearing mice treated with doxorubicin, MDSC-derived sEVs directly accelerate the proliferation and metastasis of tumor cells, and this is mediated by miR-126a (151). Furthermore, sEVs from MDSCs mediate the induction of angiogenesis, enhance Th2 cell responses, and inhibit T cell proliferation, thereby promoting an immunosuppressive microenvironment (151). Additional research with these MDSC-derived sEVs will shed light on their interactions with other tumor-fighting immune cells/sEVs and as such, their influence on anti-cancer immunotherapeutic response. It will also be interesting to explore the possibility of harnessing the immune suppressive properties of MDSC-derived sEVs as a therapeutic option in autoimmune diseases to mitigate the uncontrolled immune responses.

### sEVs Derived From Tumor-Associated Macrophages (TAMs)

In the tumor microenvironment TAMS are the most represented population of immune cells (152). TAMS promote angiogenesis, invasion and metastasis, and modulate immunosuppression (153–156). There is emerging evidence that TAMS are able to produce sEVs (157) which impact both malignant and non-malignant cells in the TME. TAMS have essentially two opposing phenotypes; M1 which is anti-tumorigenic and M2, which is pro-tumorigenic. It appears that the immunosuppressive activity of sEVs secreted from TAMs is predominantly from M2 polarized macrophages. sEVs derived from M2 TAMS promote tumor cell migration and invasion (158, 159). TAM-derived sEVs have been reported to mediate the interaction of TAMS with T cells in the TME through the transfer of specific miRNAs, which induces a Treg/Th17 imbalance and facilitates tumor progression and metastasis in ovarian cancer (160). Furthermore, TAM-derived sEVs confer therapeutic resistance to ovarian cancer cells, again via the transfer of miRNAs (161). In contrast to these findings, in preclinical models of colon and breast cancer, TAM-sEVs have been shown to have a molecular profile more indicative of an M1 polarization signature, and their cargo is enriched in markers of inflammation and lipid metabolism (162). These sEVs promote T cell proliferation and activation, and thus may have the potential to stimulate anti-tumor immunity (162). The ability of TAM-derived sEVs to potentiate the immunosuppressive function of TAMS or promote anti-tumor immunity may thus be context- and tumor type-dependent, and will require additional studies to clarify. Nevertheless, targeting TAM-derived sEVs, or the specific miRNAs that are critical for the immunosuppressive activities of TAMs, may be a potential therapeutic approach to facilitate current therapeutic strategies.

### sEVs Derived From γδ T Cells

γδ T cells are a distinct subgroup of T cells containing T cell receptors γ and δ (163, 164). These cells represent only a minor lymphocyte population making up ~0.5–16% of total CD3^+^ T cells in the peripheral blood, but they predominate in the skin and intestine (165). Due to their unique biology and well-established role in cancer immunosurveillance, γδ T cells are gaining considerable attention. γδ T cells have a somewhat dichotomous function in that they can be both anti-tumorigenic and pro-tumorigenic.

Several features of γδ T cells makes them potential suitable candidates for anti-tumor immunotherapy: (i) their ability to recognize tumor antigens independently of MHC restriction and co-stimulation (166); (ii) production of effector cytokines (TNF-α and IFN-γ) and conferring cytotoxicity against tumor cells both directly and indirectly by stimulating macrophages and DCs (167–169); and (iii) activated γδ T cells acquire the phenotype of antigen presenting cells (APCs) and induce CD4^+^ and CD8^+^ T cell proliferation and cytotoxicity (170). The pro-tumorigenic properties of γδ T cells are largely driven by IL-17A, whose expression in γδ T cells is increased in preclinical models for several cancers (171–176). IL-17A from γδ T cells binds to IL-17 receptors and promotes cancer progression via several downstream effects on malignant cells as well as other immune cells (177). γδ T cells stimulate endothelial cells to promote angiogenesis (171), promote the recruitment of pro-angiogenic macrophages to tumors (175), and are one of the main chemoattractants for the recruitment of MDSCs (178). These pro-tumorigenic properties of γδ T cells translate into the clinic since IL-17-producing γδ T cells are associated with poor survival in several cancers (179–181).

Currently γδ T cell-derived sEVs are understudied. Since sEVs derived from other immune cells exhibit the characteristics and carry the cargo of their parental cells (109, 182, 183), it is reasonable to assume that sEVs derived from γδ T cells will inherit the characteristics and functions of the parental cells. Indeed, typical sEVs can be derived from expanded γδ T cells *ex vivo* and these sEVs express several of the cytotoxic markers (including NKG2D, FasL, TNF-α, and IFN-γ) of the parental cells and are able to inhibit tumor growth (184). Interestingly, overexpression of miR-138, a miRNA with tumor suppressor and immunoenhancing properties in γδ T cells resulted in a concomitant increase of miR-138 in sEVs derived from these γδ T cells. These miR-138 rich sEVs effectively stimulate anti-tumor immunity and exhibit a more potent cytotoxic effect on tumor cells (184). Since γδ T cell-derived sEVs inherit the cytotoxicity capacity of the parental cells, this raises the possibility that these sEVs can serve as an effective drug delivery system.

The pro-tumorigenic properties of γδ T cells are largely IL-17 driven. IL-17-producing γδ T cells are rarely found in healthy individuals, however these cells accumulate in inflammatory disease, such as cancer (179, 180, 185, 186). This may, in part, explain the lack of studies on the role of γδ T cell-derived sEVs on tumor growth and immunosuppression. To the best of our knowledge, there have been no reports on the immunomodulatory effects of IL-17-expressing sEVs from γδ T cells in cancer. Of note though, IL-17-containing sEVs were found at much higher levels in patients with moderate to severe psoriasis compared to those with mild psoriasis (187), suggesting that IL-17-containing sEVs may correlate with disease progression. Unfortunately, the cell type of origin for these IL-17-containing sEVs is unknown. The exact role of IL-17-expressing γδ T cell-derived sEVs in cancer has yet to be determined, but one can predict that IL-17 containing sEVs from γδ T cells would have a similar effect on the tumor microenvironment as the parental cells. Further investigation is required to determine the exact impact of these IL-17-expressing sEVs on the tumor microenvironment and immune modulation.

### sEVs Derived From T Regulatory Cells (Tregs)

Tregs are an integral component of the adaptive immune system that contribute to maintaining immune tolerance and preventing autoimmune disease (188, 189). Tregs are a highly immunosuppressive subset of T cells that are characterized by the expression of the transcription factor FOXP3 (190–192). Tregs are recruited to the TME where their interaction with other immune cells creates an immunosuppressive environment (193), and are recognized as a major cause of reduced efficacy or failure of cancer immunotherapy (194). Tregs exert their immunosuppressive activity through several mechanisms: (1) Tregs consume excess amounts of IL-2, thereby limiting the availability of this cytokine to effector T cells (195); (2) Tregs suppress APC function through constitutive expression of CTLA4, thereby inhibiting the activation of effector T cells (196, 197); (3) Tregs express immunosuppressive cytokines such as TGF-β, IL-10, and IL-35 (198–201); (4) Tregs are instrumental in the conversion of ATP to the immunomodulatory metabolite adenosine which prevents T cell activation (202); and (5) they secrete granzyme and perforin to destroy effector cells (203).

Tregs also secrete sEVs which may arguably be critical for the immunosuppressive activity of the parental cells. Treg-derived sEVs inhibit CD8^+^ CTL responses and anti-tumor activity by suppressing T cell proliferation, modification of APCs and through CD73-mediated production of adenosine (204–206). Of particular importance in Treg-derived sEV immunomodulation is the transfer of miRNA from Tregs to target cells, which appears to be central for Treg function. sEV mediated transfer of Let-7d miRNA from Tregs to Th1 cells suppresses Th1 cell proliferation and IFN-γ production (207). Similarly, sEV-mediated transfer of miR-150-p and miR-142-3p from Tregs to DCs induced a more tolerogenic phenotype in the DCs characterized by an altered cytokine profile (208).

The immunosuppressive effects of Treg-derived sEVs in cancer are clearly detrimental, however, in other settings these effects may prove useful. For example, in the case of organ transplantation, Tregs can be engineered to express specific proteins and miRNAs, which accumulate in sEVs from these modified Tregs. These modified sEVs inhibit T cell alloreactivity and induce immune tolerance in transplantation and may be a useful therapeutic option to manipulate the immune system in patients undergoing organ transplantation (209).

The study of Treg-derived sEVs is relatively new. There is no doubt, however, that Treg-derived sEVs contribute significantly to Treg cell function and therefore identifying and targeting the immunosuppressive cargo of Treg-derived sEVs may serve as a potential therapeutic option for cancer. Conversely, since the cargo of sEVs can be altered by genetically modifying the parental cells, “designer” Treg-derived sEVs have the potential to act as therapeutic agents in patients with autoimmune disorders or who are receiving transplants.

## Bioengineering of Immune Cell-derived sEVs

Immune cell-derived sEVs can either activate or suppress the immune responses and these contrasting outcomes are dictated by (1) composition of the cargo, (2) activation and maturation status of the immune cells, (3) identity of target cells, and (4) disease setting and host microenvironment. T cell- and activated NK cell-derived sEVs are reported to express death ligands FasL, Apo2L, and TRAIL. These death ligands, expressed in a membrane-bound form, can interact with death receptors and induce apoptosis or immune tolerance in target cells (e.g., autologous T cells and DCs) (210). Also, sEVs released from immature and mature DCs impart opposing effects on the antigen-specific immune responses. When compared with sEVs from mature DCs, sEVs from immature DCs express sub-optimal levels of MHC class II and co-stimulator molecules (CD80, CD40, CD86, and ICAM I) and thus, are unable to drive immunostimulatory responses, instead dampen *in vivo* immunity and promote immune tolerance (69, 211–213).

In the past decade, several research groups have focused on the study of modulation of the immunogenicity of sEVs. The cargo expressed by immune cell-derived sEVs can be manipulated to overcome the immune suppressive properties of sEVs. As mentioned earlier, sEVs from T cells activated with IL-2 or IL-12 can directly stimulate proliferation of bystander resting T cells; this activation does not require the presence of antigens or APCs (112, 113). The effects of modified dexosomal vaccine formulations including adjuvants that induce DC maturation and activation have been reported. Modified poly(I:C)- and OVA antigen-expressing dexosomes stimulate OVA-specific anti-tumor T effector responses and reduce tumor growth (79). DC-derived sEVs have also been modified to harbor the iNKT ligand α-galactosylceramide; this modification enables the dexosomes to activate the invariant natural killer T cells (iNKT) that results in stronger immune effector responses *in vivo* (78). Dexosomes from α-fetoprotein (AFP)-expressing DCs have been shown to exhibit efficient anti-tumor activity in transplantable, orthotopic and carcinogen-induced hepatocellular carcinoma (HCC) mouse models (71).

The tumor-destroying capabilities of NK cell-derived sEVs can be utilized to treat “cold tumors” that are non-T cell inflamed and contain immune suppressive cells. Blood-brain crossing capabilities of NK cell-derived sEVs can be exploited to treat brain tumors. As with the endogenous NK cells, it is possible that tumors can develop various inhibitory mechanisms to shield themselves from sEV-mediated killing. However, genetic engineering tools (e.g., blocking inhibitory receptor expression) and *ex vivo* NK cell activation can be used to reshape the cargo of the NK cell-derived sEVs and influence their immune stimulatory activity.

Several different approaches to load exogenous proteins into sEVs have been explored. Transfection of cells with exogenous transmembrane proteins such as G protein-coupled receptors (CCR7, CCR2, and CXCR4) can lead to secretion of sEVs expressing these exogenous proteins (214, 215). Such modified sEVs can be used as a valuable tool in drug development studies. B cell-derived sEVs that exogenously express C3 complement proteins can interact with G protein coupled receptors on T cells and induce immune activation (97).

A second approach to modify the protein content in sEVs involves the use of a chimeric protein consisting of the sEV surface expressed LAMP-2b protein fused to a RGV peptide obtained from a rabies virus glycoprotein. DCs are then transfected with the chimeric protein, and the sEVs obtained from these cells are incorporated with siRNA targeting mutant Huntington mRNA (216). These modified sEVs are able to cross the blood-brain barrier and are capable of actively blocking the expression of the Huntington protein in neurons *in vivo* in mice (216). However, LAMP-2b fusion proteins need to be further modified to protect against lysosomal degradation (217). One other method of loading proteins into sEVs involves the use of the C1C2 (lipid-binding) domains of the human lactadherin protein to generate protein chimeras that bind to phosphatidylserine in the sEV membranes in a non-covalent manner (218, 219). Such engineered sEVs are biologically and functionally active.

Recent studies have explored the use of artificial EVs that are pre-loaded with immunostimulatory cargo. These artificial EVs overcome at least two major short comings of primary immune cell-derived EVs: (1) Primary immune cells are difficult to isolate to 100% purity and cannot be homogenously activated (the isolated EVs are often contaminated with tolerogenic/immunosuppressive EVs); and (2) Production and expansion of large quantities of primary autologous clinical grade human cells is expensive and labor intensive. Therefore, artificial EVs, which are basically liposomes coated with immunostimulatory molecules/ligands, can be a practical and cost-effective substitute for primary immune cell-derived EVs (220).

## Discussion

sEVs can be manipulated to promote immunogenicity against cancer, and such bioengineered sEVs can function as efficient cancer vaccines. Immunostimulatory sEVs derived from immune cells, particularly from DCs, hold promise as therapeutic agents against cancer, as they initiate effective anti-tumor immunity when compared to that of other cell-free therapeutic strategies. Advantages of sEVs include high bio-availability, bio-stability, and lower costs. However, significant questions pertaining to sEVs' potential use as an immune-prophylactic agent or therapeutic for cancer treatment remain unanswered: (1) What is the precise mechanism of action? (2) Which specific cancers are sensitive to this therapeutic strategy? (3) Which membrane vesicle characteristics define immunostimulatory or immunosuppressive properties of sEVs? (4) Can sEV-mediated immunostimulatory effects be reproduced with different batches of sEV preparations? and (5) Can the stimulatory potential of immune cell-derived sEVs counter the immunosuppressive milieu created by factors/molecules secreted by tumor cells? Finding answers to these questions will be key for the success of sEV-based anti-cancer therapies. Currently it is not known exactly what fraction of the total circulating sEVs are immune cell-derived, which would indicate the relative importance of these immune cell-derived sEVs to disease pathology. However, immune cell-derived sEVs play a critical role in tumor progression either negatively or positively. Determining the exact fraction of immune cell-derived sEVs in the total population of circulating EVs will require extensive and accurate characterization of the total circulating sEV population.

Thus, while sEVs have been shown to activate anti-tumor immune responses in pre-clinical studies, clinical data in cancer patients have yielded only modest benefits. Recent advances in deciphering the molecular composition and functional characteristics of immune cell-derived sEVs can lead to next-generation agents with potential for use in anti-cancer immunotherapy. sEVs derived from specific cell sources, such as the MDSCs, Tregs and TAMS may contain additional molecules which can be harnessed as targets for inducing greater levels of host immune responses against cancers.

## Author Contributions

KY, CL, and HD wrote the paper. JE edited the manuscript and contributed to the scientific discussion in this manuscript. All authors approved the manuscript for publication.

## Conflict of Interest

The authors declare that the research was conducted in the absence of any commercial or financial relationships that could be construed as a potential conflict of interest.
